# DEPRESSION AMONG MIDDLE-AGED AND OLDER INFORMAL CAREGIVERS IN 19 COUNTRIES: A CROSS-NATIONALLY HARMONISED STUDY

**DOI:** 10.1093/geroni/igae098.1665

**Published:** 2024-12-31

**Authors:** Nan Jiang, Jose Pagan, Bei Wu, Yan Li

**Affiliations:** Tsinghua University, Beijing, Beijing, China (People’s Republic); New York University, New York, New York, United States; New York University, New York, New York, United States; Shanghai Jiao Tong University School of Medicine, Shanghai, Shanghai, China (People’s Republic)

## Abstract

The health of the caregivers is crucial to sustain informal care provision, while depression is an important health risk concept. In this study we aim to conduct multi-country, population-based studies on caregivers’ depression and examine the association of caregiving with depression in 19 countries. We included 142,741 adults aged ≥ 45 years in our sample. Depression was measured using established cut points in shortened Center for Epidemiologic Studies Depression (CES-D) or EURO-D scales. We conducted multivariable logistic regression analyses to explore the association of the past 12-month caregiving with depression. Over 30% of participants in our sample were engaged in caregiving with particularly high rates observed in Denmark, China, France, and Sweden. After controlling for potential confounders, caregivers had a significantly higher risk of having depression (OR = 1.13, 95% CI = 1.02-1.25) compared to non-caregivers. The associations were stronger in China, India, South Korea, Spain, and the United Kingdom than in other countries. Our findings suggest that urgent attention must be given to developing strategies that address the unique challenges faced by caregivers, fostering their well-being while maintaining high-quality care standards. Future interventions and policies need to aim at mitigating the mental health burden experienced by caregivers and ensuring sustainable and effective care support.

